# Supplementary material for: Replication, Communication, and the Population Dynamics of Scientific Discovery
Source: PLoS One. 2015 Aug 26;10(8):e0136088. doi: 10.1371/journal.pone.0136088 (PMC4550284; doi:10.1371/journal.pone.0136088)
Supplement: S1 Text — (PDF) [file pone.0136088.s001.pdf]

SI TEXT
REPLICATION, COMMUNICATION, AND THE POPULATION DYNAMICS OF SCIENTIFIC DISCOVERY

RICHARD MCELREATH[1,2] AND PAUL SMALDINO[1]

Supplemental. Date: April 6, 2015

1. Derivation of full model with random replication

Let $f_{\text{T},s} = n_{\text{T},s}/n$ be the frequency of true hypotheses with tally s. Under the assumptions and definitions supplied in the main text, the full recursion for $n'_{\text{T},s}$ is given by:

$$n'_{\text{T},s} = n_{\text{T},s} + anr\big(-f_{\text{T},s}(c_{\text{R}+}(1-\beta) + c_{\text{R}-}\beta) + f_{\text{T},s-1}(1-\beta)c_{\text{R}+} + f_{\text{T},s+1}\beta c_{\text{R}-}\big) \tag{1}$$

for s not equal to 1 or -1. In those cases, there is an additional term. For $s = 1$:

$$\begin{aligned} n'_{\text{T},1} &= n_{\text{T},1} \\ &\quad + anr\big(-f_{\text{T},1}(c_{\text{R}+}(1-\beta) + c_{\text{R}-}\beta) + f_{\text{T},0}(1-\beta)c_{\text{R}+} + f_{\text{T},2}\beta c_{\text{R}-}\big) \\ &\quad + an(1-r)b(1-\beta) \end{aligned} \tag{2}$$

The $an(1-r)b(1-\beta)$ term accounts for inflow of novel positive findings, all of which are communicated. For $s = -1$:

$$\begin{aligned} n'_{\text{T},-1} &= n_{\text{T},-1} \\ &\quad + anr\big(-f_{\text{T},-1}(c_{\text{R}+}(1-\beta) + c_{\text{R}-}\beta) + f_{\text{T},-2}(1-\beta)c_{\text{R}+} + f_{\text{T},0}\beta c_{\text{R}-}\big) \\ &\quad + an(1-r)b\beta c_{\text{N}-} \end{aligned} \tag{3}$$

The $an(1-r)b\beta c_{\text{N}-}$ term accounts for inflow of novel negative findings, only $c_{\text{N}-}$ of which are communicated. Recursions for false hypotheses can be derived just by substitution of variables: $b \to 1-b$ and $1-\beta \to \alpha$.

These recursions implicitly define the population growth recursion for n:

$$n' = n + an(1-r)\big(b(1-\beta+\beta c_{\text{N}-}) + (1-b)(\alpha + (1-\alpha)c_{\text{N}-})\big) \tag{4}$$

This just indicates that the population of published hypotheses grows proportional to the innovation rate, $1-r$, and the rates at which true and false hypotheses respectively produce positive and negative findings, as well as the rate at which negative findings are communicated.

[1]Department of Anthropology, UC Davis, One Shields Avenue, Davis CA 95616
[2]Center for Population Biology, UC Davis
E-mail address: mcelreath@ucdavis.edu.

2. Beyond "true" and "false"

Above we noted that recursions for false hypotheses can be derived just by substitution of variables: $b \to 1 - b$ and $1 - \beta \to \alpha$. In other words, true and false hypotheses are differentiated only by the rate at which they appear in new investigations and their respective probabilities of producing positive findings. This also means it is straightforward to expand the model to additional epistemic states, as "true" and "false" really just more more and less correct. For example, small, medium, and large effect sizes could be represented by three states, each with its own base rate and probability of producing a positive result. The derivation would remain the same, but an additional set of steady-state solutions would appear.

3. Steady-state solutions

We have analyzed this model using a variety of methods. First, we solved the model analytically for every structure except for targeted replication (to be defined later). Second, when analytical solution was not possible, we solved the model numerically. Third, we studied the model under both deterministic and stochastic simulations, written independently by both authors in different programming languages. All forms of analysis yield identical results.

The model above can be solved directly, in one of two ways. First, it can be solved exactly by bounding tallies within a minimum and maximum (using either absorbing or reflecting boundaries) and then solving the system of simultaneous equations for values of the state variables $f_{i,s}$ for $i \in \{\mathrm{T}, \mathrm{F}\}$. This approach is probably the most straightforward. Second, it can be solved to any level of approximation desired by iteratively solving the system of equations outward from $s = 0$.

Both approaches yield solutions that take the form of closures of infinite geometric series expressions. Using these solutions, we found the unbounded infinite series solution based upon intuition—*ansatz* is what our mathematics instructors used to call it. Since the solutions from the brute-force approach looked like closures of infinite series, and the simulation results produced what resembled a mixture of geometric series, we guessed the underlying limiting distribution. We then verified our *ansatz* solution by plugging it back into the recursions and also by comparing it to numerical results and our previous solutions. Finally, we induced the infinite series representation by constructing Taylor series expansions of the closed series expressions, yielding the sequential terms of the solution expression in the next section.

3.1. **Full communication solution.**

Here we repeat the simplest such solution from the main text and then motivate its justification. The steady state proportion of hypotheses that are both true and have tally s, when all findings are communicated, is given by:

$$\hat{p}_{\mathrm{T},s} = b(1-r) \sum_{m=1}^{\infty} r^{m-1} K\big(m, (m+s)/2\big) (1-\beta)^{\frac{1}{2}(m+s)} \beta^{\frac{1}{2}(m-s)} \tag{5}$$

where $K(m, (m+s)/2)$ is the number of ways to get $(m+s)/2$ positive findings in m investigations of the same hypothesis. This is simple the binomial chooser, but implicitly evaluating to zero whenever $(m+s)/2$ is not an integer. Since s is the difference between the number of positive and negative findings, this multiplicity accounts for the number of paths by which an hypothesis can be studied m times and end up with a tally s. The remaining terms leading

with $1-\beta$ and β are just the probabilities of getting $(m+s)/2$ positive findings and $(m-s)/2$ negative findings, respectively.

Here's how to motivate the above solution. For any given tally s, there are an infinite number of histories by which it could have ended up with that tally.

- Consider tally $s = 1$, for example. If the hypothesis is true, it could end up most simply at $s = 1$ with just one initial positive finding. This happens with probability $(1 - r)b(1 - \beta)$, indicating innovation times base rate of true hypotheses times the probability of an initial positive finding.
- Similarly, if instead the hypothesis has been studied twice, which happens $(1 - r)br$ of the time, the number of ways it could end up with $s = 1$ is exactly zero, and the multiplicity handles this by assigning $K(2, (2 + 1)/2) = 0$.
- For three studies, there are $K(3, 2) = 3$ ways $s = 1$ could happen. Represented as sequences of positive and negative findings, these are: (1) $++-$, (2) $+-+$, and (3) $-++$. The probability of any one of these is $(1 - \beta)^2\beta$, and the probability that an hypothesis is true and has been studied three times is $(1 - r)br^2$.

The pattern here generalizes so that the total probability is just:

- the sum over number of studies on an hypothesis from $m = 1$ to $m = \infty$ of the probability the hypothesis was studied m times, given by $(1 - r)r^{m-1}$
- times the number of ways it could end up with a tally s in m steps, given by $K(m, (m+s)/2)$
- times the probability of getting $(m + s)/2$ positive and $(m - s)/2$ negative findings.

Writing down this summation and factoring out the common term $b(1 - r)$ completes the expression.

This steady-state solution obviously assumes that there has been an infinite amount of research time, such that every m can be realized. In practice, since the sequence is geometric in r, the probabilities of higher values of m decline very rapidly and simulations confirm that steady-state is reached quite rapidly, as long as the replication rate r is not close to $r = 1$.

More importantly we think, these solutions are never meant to describe actual science, but rather to allow us to reason about causal forces in actual science. So the steady state expressions are important even if, as in many real dynamical system, they are never exactly realized. For example, problems in evolutionary theory are routinely solved by asking what happens on the infinite time horizon. Such solutions have been incredibly useful, despite the fact that no real population or environment is stationary enough to make the exercise literally sensible.

3.2. **Arbitrary communication solution.** When communication parameters are allowed to be less than one, the above strategy generalizes directly, but does become complex. The expressions get much more complex, because now the infinite series is over multinomial probabilities of three possible outcomes at each replication investigation of an hypothesis: (1) positive and communicated, (2) negative and communicated, or (3) not communicated. In addition, when findings are not always communicated, then the effective activity rate changes, making other probabilities conditional on observable activity. Still, these solutions can be derived both by the logic to follow or by brute-force solution of the system of recursions. Solving the system of recursions does allow for easily defining reflecting or absorbing tally boundaries, which may be appealing in some contexts. The combinatoric solution to follow assumes unbounded tallies. Solutions in the bounded and unbounded cases are

nearly identical, for all scenarios considered in the main text. The Mathematica notebooks in the supplemental materials present code for both types of solution.

We present the solutions here as a sequence of conditional probabilities, as we've found this form easier to interpret than the general multinomial form. Therefore they provide more insight. Specifically, we decompose the multinomial probabilities into a binomial series for observed/unobserved investigations of a hypothesis and a binomial series for positive/negative findings conditional on being observed. The solutions take the form:

$$\hat{p}_{\mathrm{T},s} = \Pr(\mathrm{T}) \Pr(\text{activity}) \Pr(\text{new}|\text{activity}) \big((1-\beta) \Pr(s|+) + \beta c_{\mathrm{N}-} \Pr(s|-) \big) \tag{6}$$

Where:

$$\Pr(\mathrm{T}) = b \tag{7}$$

$$\Pr(\text{activity}) = r + (1-r)\big(b((1-\beta) + \beta c_{\mathrm{N}-}) + (1-b)(\alpha + (1-\alpha)c_{\mathrm{N}-})\big) \tag{8}$$

$$\Pr(\text{new}|\text{activity}) = \frac{(1-r)\big(b((1-\beta) + \beta c_{\mathrm{N}-}) + (1-b)(\alpha + (1-\alpha)c_{\mathrm{N}-})\big)}{\Pr(\text{activity})} \tag{9}$$

The probabilities $\Pr(s|+)$ and $\Pr(s|-)$ give the probabilities of tally s averaging over number of investigations m and un-communicated findings u, beginning with either a positive finding or a negative finding, respectively. This conditioning is necessary because a tally s can be reached by different paths once communication is partial. These probabilities are given by:

$$\Pr(s|+) = I_1(s) + \sum_{m=1}^{\infty} \sum_{u=0}^{m} R^m \Pr(u|m) S(s-1|m-u) \tag{10}$$

$$\Pr(s|-) = I_{-1}(s) + \sum_{m=1}^{\infty} \sum_{u=0}^{m} R^m \Pr(u|m) S(s+1|m-u) \tag{11}$$

where $I_a(b)$ is a function that returns 1 when $a = b$ and zero otherwise and $R = r/\Pr(\text{activity})$ is the probability of replication, conditional on activity as defined earlier. The term $\Pr(u|m)$ gives the probability of u un-communicated findings in m investigations, defined as:

$$\Pr(u|m) = \frac{m!}{u!(m-u)!} q_\circ^u (1-q_\circ)^{m-u} \tag{12}$$

where

$$q_\circ = (1-\beta_\mathrm{R})(1-c_{\mathrm{R}+}) + \beta_\mathrm{R}(1-c_{\mathrm{R}-}) \tag{13}$$

is the probability a replication finding is un-communicated, averaging over positive and negative findings. Finally, the function $S(z|n)$ provides the probability that a sequence of length n communicated replication findings producing a difference z between positive and negative replications. It is defined as:

$$S(z|n) = \begin{cases} I_0(z) & \text{if } n = 0 \\ K(n, (n+z)/2) q_+^{(n+z)/2} (1-q_+)^{(n-z)/2} & \text{if } n > 0 \end{cases} \tag{14}$$

where $K(a, b)$ is again the binomial chooser function, but evaluating to zero when b is not an integer, and:

$$q_+ = \frac{(1-\beta_\mathrm{R})c_{\mathrm{R}+}}{1-q_\circ} \tag{15}$$

which is the probability of a positive replication, conditional on the replication finding being communicated.

4. Approximate conditions for reduced communication

We argue in the main text that full communication is rarely optimal, from the perspective of precision. Consider the full communication context: $c_{\mathrm{N-}} = c_{\mathrm{R-}} = c_{\mathrm{R+}} = 1$. For small b ($b^2 \approx 0$) and small r ($r^3 \approx 0$), precision as defined in the main text is improved by reducing communication parameters under the following conditions:

- $c_{\mathrm{N-}} < 1$ when $\alpha < \beta$ (easy to satisfy)
- $c_{\mathrm{R-}} < 1$ when $\alpha > 0.5$ (hopefully not satisfied)
- $c_{\mathrm{R+}} < 1$ when $\beta - \alpha \leq 1/4$

These conditions are derived by first defining precision at $s = 1$, which is most conservative precision to investigate, because it benefits the least from replication, and higher tallies always have higher precision than $s = 1$. So improvements at $s = 1$ cascade upwards to higher tallies. Let PPV_1 be the precision at $s = 1$. Then the first condition is proved by computing the derivative $\partial \mathrm{PPV}_1 / \partial c_{\mathrm{N-}}$, evaluated at full communication parameter values. Then Taylor expand the result simultaneously by second-order around $r = 0$ and by first-order around $b = 0$. Neglecting terms of order $O(b^2)$ and $O(r^3)$ and higher:

$$\frac{\partial \mathrm{PPV}_1}{\partial c_{\mathrm{N-}}} \approx -r^2 \frac{1-\beta}{\alpha} b(\beta - \alpha)(1 - \beta - \alpha)(5 - 6\alpha) \tag{16}$$

which is negative unless $\alpha > \beta$. Thus suppressing some initial negative findings is favorable, provided the base rate is small and replication is not too common. We think most scientific fields satisfy these conditions, but reasonable people can and do disagree on that point.

In contrast, suppressing negative replications is unlikely to help. By the same strategy, but this time differentiating with respect to $c_{\mathrm{R-}}$:

$$\frac{\partial \mathrm{PPV}_1}{\partial c_{\mathrm{R-}}} \approx rb \frac{1-\beta}{\alpha}(1 - \beta - \alpha)(1 + 2r(\beta - \alpha)) \tag{17}$$

which is guaranteed positive, indicating that $c_{\mathrm{R-}} = 1$ is favored, when $\alpha \leq 0.5$, because by assumption $1 - \beta > \alpha$.

The third condition is derived similarly:

$$\frac{\partial \mathrm{PPV}_1}{\partial c_{\mathrm{R+}}} \approx -br \frac{1-\beta}{\alpha}(1 - \beta - \alpha)(1 - 4r(\beta - \alpha)) \tag{18}$$

The last term is the one in play. For the above to be negative, it is required that:

$$r < \frac{1}{4} \frac{1}{\beta - \alpha} \tag{19}$$

And this is guaranteed when $\beta - \alpha \leq 1/4$.

References
